# Vibration related symptoms and signs in quarry and foundry workers

**DOI:** 10.1007/s00420-021-01660-8

**Published:** 2021-02-13

**Authors:** Lars Gerhardsson, Christina Ahlstrand, Per Ersson, Per Jonsson, Ewa Gustafsson

**Affiliations:** Department of Occupational and Environmental Medicine, School of Public Health and Community Medicine, Institute of Medicine, Sahlgrenska Academy, University of Gothenburg, Gothenburg, Medicinaregatan 16A, Box 414, SE-405 30, Gothenburg, Sweden

**Keywords:** Quarry, Foundry, Hand-arm vibration syndrome (HAVS), Vibration exposure

## Abstract

**Purpose:**

The development of vascular and neurosensory findings were studied in two groups of long-term exposed quarry and foundry workers with different vibration exposures, working conditions and work tasks.

**Methods:**

The study included 10 quarry workers (mean age 43 yrs., mean exposure time 16 yrs.) and 15 foundry workers (35 yrs.; 11 yrs.) at two plants in Sweden. All participants completed a basic questionnaire and passed a medical examination including a number of neurosensory tests, e.g. the determination of vibration (VPT) and temperature (TPT) perception thresholds as well as a musculoskeletal examination of the neck, shoulders, arms and hands.

**Results:**

A high prevalence of neurosensory findings (40%) was found among the quarry workers. Both groups, however, showed a low prevalence of vibration white fingers (VWF). Foundry workers showed significantly better sensitivity than quarry workers for all monofilament tests (*p* ≤ 0.016), TPT warmth in dig 2 (*p* = 0.048) and 5 dexter (*p* = 0.008), and in dig 5 sinister (*p* = 0.005). They also showed a better VPT performance in dig 5 dexter (*p* = 0.031).

**Conclusions:**

Despite high vibration exposure, the prevalence of VWF was low. The high prevalence of neurosensory findings among the quarry workers may depend on higher A(8) vibration exposure and higher exposure to high-frequency vibrations. An age-effect and exposure to cold could also be contributing factors. The nervous system seems to be more susceptible to high-frequency vibrations than the vascular system. For neurosensory injuries, the current ISO 5349-1 standard is not applicable.

## Introduction

Vibration-related injuries are a major problem in most countries. The exposure can cause vibration white fingers (VWF), neurosensory disturbances such as numbness and tingling, reduced grip strength and decreased manual dexterity (Gemne 1997; Heaver et al. 2011; Gerhardsson et al. 2013). A dose–response relationship between exposure to hand transmitted vibration and the development of neurosensory disorders and reduced work ability has been reported by Bovenzi et al. (2015). Neurosensory sequelae after a local freezing injury affecting thermal and vibration perception thresholds may last more than 4 months after the cold injury (Carlsson et al. 2014). In a systematic review and meta-analysis by Nilsson et al. (2017), the authors found that neurosensory injuries occur with a 3-time factor shorter latency than Raynaud’s phenomenon. Long-term effects of hand-arm vibration on thermotactile perception thresholds has been presented by Lundstrom et al. (2018). A 22-year follow-up study of vibration-exposed sheet metal workers showed a tendency towards irreversible hand numbness and finger pain in workers with HAVS (Hand-arm vibration syndrome; Aarhus et al. 2019). Finger and hand pain occur quite often in HAVS subjects (House et al. 2016). In a long-time follow-up study by Aarhus et al. (2018), continued vibration exposure was found to worsen the white finger symptoms. The cost for vibration injuries for the individual, the company and the society are substantial.

Two highly exposed occupations are quarry and foundry workers. They work with different types of tools and have different vibration exposures. Kákosy et al. (2003) studied vascular, neurological and musculoskeletal symptoms in 95 foundry workers using chipping hammers and grinders. The first indications of vibration-related symptoms and signs appeared after 7 years of exposure. In the study group, 79% reported vascular symptoms, and 65% neurosensory symptoms. Lesions of the peripheral nerves in the arms were noted among 41 subjects, and the carpal tunnel syndrome in 21 subjects. The most effective preventive action according to the authors was a reduction of the daily exposure time.

At a Finnish foundry, all workers (*N* = 12) developed vibration white fingers after starting to use grinding wheels made of zirconium-corundum mixed with silica instead of corundum (Starck et al. 1983). The mean latency time for the workers was no longer than 10 months. None of the workers had shown signs of vibration white fingers before the introduction of the new grinding wheels. All workers complained about numbness in fingers and hands, which disturbed their sleep. Their vibration detection thresholds were significantly raised at all frequencies compared with a reference group. The authors found that the new wheels added 12 dB (fourfold increase) to the levels of the old wheels in the frequency range 25–160 Hz. They also suspected that increased exposure to transient and high-frequency vibrations that was caused by the new grinding wheels could contribute to the rapidly developing adverse health effects.

Agate (1949) studied men and women in two shops working with polishing and grinding castings made either of steel or of a duralumin type of alloy. In this survey, 70% of the 233 exposed males and 47% of the 45 exposed females developed vibration white fingers.

In an Italian study, Bovenzi et al. (1987) investigated a group of vibration-exposed foundry workers (mean age around 40 yrs), who were compared with a reference group of similar age. The latter group performed heavy manual work without vibration exposure. The vibration-exposed workers showed a higher prevalence of musculoskeletal symptoms, e.g. arthralgias of the wrist and elbows, muscle pain and lower muscular force compared with the reference group.

A century ago, Hamilton (Hamilton 1918) studied the effect of air hammers on the hands of stonecutters, mainly working in limestone. The pneumatic hammers delivered about 3000–3500 strokes per minute. The handle was held by the right hand and the chisel by the left hand, which thus received the highest vibration exposure. The author described several workers with vibration white fingers, which in most cases were linked to the exposure to the air hammers. Sixty years later, Taylor et al. (1984) examined 30 remaining stonecutters from this area, showing a very high prevalence of both VWF (60–80%) and neurosensory findings (50–75%).

Stonedrillers and stonecutters/chippers in the travertine quarries in Tuscany, Italy, also showed a high prevalence of VWF and neurosensory disturbances. In one study, Bovenzi et al. (1988) observed a prevalence of VWF of 36% with a median latent period of 10 years. In another study, Bovenzi et al. (1994) found a prevalence of VWF among stone workers of approximately 30%, and 40% for neurosensory symptoms.

## Aims

The aim was to study the differences in vascular and neurosensory findings in two groups of long-term exposed quarry and foundry workers with different vibration exposures, working conditions and work tasks.

## Material and methods

The present study is part of a large ongoing Swedish research project named Zero vibration injuries (30 partners), with the intention to achieve a considerable reduction of the vibration exposure from handheld power tools. In the present study, the development of HAVS was investigated in two groups of highly vibration-exposed workers with different work tasks and working conditions.

### Study population

The study included 10 male quarry workers and 15 male foundry workers at two plants in the middle of Sweden. It comprised all workers that used chisel and drilling machines at the quarry and chisel machines at the foundry. The studied work sites at the quarry and at the foundry were rather small, so there was a close contact between the workers and the management was highly motivated. We found a common interest to participate in the study, which gave this high participation rate.

The mean-age among the quarry workers was 43.2 ± 9.2 yrs and the mean exposure time was 15.7 ± 13.3 yrs. The corresponding figures for the foundry workers were 34.7 ± 12.5 yrs and 11.3 ± 8.5 yrs, respectively.

All participants signed a written consent and completed a basic questionnaire with questions about e.g. work and medical history, exposure time, type of vibrating tools and hand-arm vibration symptoms. The study was approved by the ethical committee at the University of Gothenburg.

### Measurements of vibration exposure

The vibration exposure estimation was based on the information provided by the participants in the questionnaires including the time of exposure to vibrating tools (months/years, daily exposure time in minutes), work tasks, type of vibrating tools, and the perceived magnitude of vibration exposure in the right and left hand, respectively.

Information about the type of tools used at each work-station was obtained on site. Vibration levels were calculated by combining CE declared values with values from the EU handbook (2002) and measurements at the two sites made by technicians from the Research Institute of Sweden (RISE).

The combined data from the questionnaires, the additional measurements at the plant and the inventory list formed the basis for the individual vibration exposure estimations.

### Medical examinations

An experienced physician performed the medical examination. The tests included 2 point discrimination (2-PD), tuning fork, Semmes–Weinstein’s monofilament test (5-filament kit), sensitivity to pain (needle) and tests of handgrip strength (Jamar) and finger muscle strength tests (pinch grip and 3-Chuck-grip) A biomedical analyst performed the neurosensory tests that included the determination of thermal (TPT) and vibration perception thresholds (VPT). The symptoms and signs were staged according to the Stockholm Workshop Scale (SWS). The staging of the vascular syndrome goes from 0 to 4 and from 0 to 3 SN for neurosensory symptoms. A higher staging means a more serious disease. The participants were told to avoid vibration exposure during the day of the measurement as well as intake of tobacco and coffee at least one hour before the medical tests.

Nine of the quarry workers were examined by an experienced physiotherapist that performed a musculoskeletal examination of the neck, shoulders, arms and hands. One worker was not available for examination.

### Thermal thresholds

To determine thermal perception thresholds, a unidirectional stimulation technique was used that is based on a commercially available test instrument with a Peltier element-based thermode of 25 × 50 mm (Termotest®; Somedic Sales AB). The starting temperature was 32 degrees for both cold and warmth and the pulps of digits 2 and 5 bilaterally were tested. To make the testing more comfortable, the forearm and the wrist of the participant were supported. The perception thresholds to non-painful cold and warmth, respectively, were obtained by delivering six cold stimuli, followed by six warm stimuli in random order, at a rate of 1 °C/sec. At the first feeling of cold and warmth, the subject was instructed to press the button of a handheld switch. Then, the temperature decreased or increased by 1 °C per second until the subject released the response button. The test procedure was repeated another five times. The mean of the last four assessments for cold and warmth on the finger pulps was registered as the cold or warmth perception thresholds.

### Vibrotactile measurements

The ascending-descending method of limits was used to deliver sinusoidal vibrations to the pulps of digits 2 and 5 bilaterally. The equipment used was the VibroSense Meter® system (Vibrosense Dynamics, Malmö, Sweden). The testing covered sinusoidal frequencies at seven frequencies (8 Hz, 16 Hz, 32 Hz, 64 Hz, 128 Hz, 256 Hz, and 512 Hz) all transmitted to the finger through a vibration probe with a diameter of 4 mm. The contact force between the probe and the finger was 1 N. Before the start of the test, the finger temperature had to reach + 28 °C. To facilitate the testing, the wrist and the forearm of the participant were supported. The vibration magnitude increased until the patient could feel the vibration in the tip of the finger. The participant then pressed the response button, after which the vibration magnitude decreased until the subject released the response button. Then the vibration amplitude started to rise again. The rate of change of the vibration amplitude was 3 dB/s and for each frequency there were six reversals. Thereafter, the testing automatically continued to the next frequency.

All results were age-corrected (Lindsell and Griffin. 2002; Seah and Griffin. 2008) by comparison with values from a reference population supplied by the manufacturer of the device. All participants used ear protective devices to exclude the noise from outdoor and indoor sources. The outcome measure was a sensibility index (SI), which was calculated by dividing the integrated area under the curve from the patient with the corresponding integrated area for the reference population, which was supplied by the manufacturer of the instrument. An SI-index < 0.8 was interpreted as an abnormal response.

Measurements of VPTs have shown a good to excellent reliability in studies of vibration-exposed workers (Gerhardsson et al. 2014). An excellent reliability has also been noticed in VPT-determinations in patients with diabetic neuropathy, ICC > 0.94 (van Deursen et al. 2001).

### Hand grip force

A Baseline® Hydraulic Hand Dynamometer (Fabrication Enterprises Incorporated, New York, NY, USA) in position number 2 was used to estimate the handgrip force. The mean of three measurements was used as the handgrip strength in the right and left hand, respectively.

For the measurements of finger muscle strength a mechanical pinch gauge (PG-60; North Coast Medical, San José, CA, USA) was used (Mathiowetz et al. 1984). The key grip strength (Pinch key) and the three-digit pinch (Pinch 3-Chuck) were measured using the mean of three measurements in each hand.

### Measurements of musculoskeletal symptoms and diagnosis

A clinical examination of musculoskeletal symptoms in neck, shoulders, elbows, and hands was performed according to the HECO-protocol (Health Surveillance in Adverse Ergonomics Conditions, MEBA in Swedish). The protocol contains separate sections for the two anatomical regions neck/shoulders and elbow/hands.

The examination consisted of questions about symptoms, tests of a range of movements, tenderness at palpation, muscle strength, sensibility, and pain or tingling at specific provocations of joints, tendons, muscles or nerves. From the protocol, the prevalence of perceived symptoms during the past 7 days, and specific diagnoses for the separate anatomical regions was established from predefined criteria (Jonker et al. 2015).

### Statistics

Normal probability plots and Levene’s test were used to test the normality of the input variables. As the majority of the variables investigated showed a skewed distribution, non-parametric statistics was used for the statistical calculations.

Differences between independent groups were evaluated with the Mann–Whitney *U*-test. Differences between paired samples (e.g. left and right hand) were analyzed with the Wilcoxon signed-rank test. The relationship between variables was investigated by calculating the Spearman rank-order correlation coefficients. P-values < 0.05 were regarded as statistically significant.

All calculations were performed with the Statistical Package for the Social Sciences (IBM SPSS Statistics 2017).

## Results

### Vibration exposure

Chisel and drilling machines were the tool categories that gave the highest vibration exposure at the quarry. The vibration levels (ISO 5349-1) were 10–18 m/s^2^ for the chisel machines and 20–22 m/s^2^ for the drilling machines. At the foundry, the highest vibration exposure came from the chisel machine. The vibration levels were 4 m/s^2^ at the maneuver handle and 20 m/s^2^ at the sleeve of the chisel machine.

The median self-estimated A(8) vibration exposure among the participants at baseline was 9.0 m/s^2^ for the quarry workers (min 0.0 m/s^2^; max 12 m/s^2^) and 6.2 m/s^2^ for the foundry workers (min 2.0 m/s^2^; max 8.9 m/s^2^) (Table 1).

**Table 1:** Vibration exposure and baseline data among quarry and foundry workers

Variable	Quarry workers *N* = 10	Foundry workers *N* = 15
Age (y)	43.2 ± 9.2	34.7 ± 12.5
Exposure time (y)	15.7 ± 13.3	11.3 ± 8.5
Median A(8) vibration exposure (m/s^2^)	9.0	6.2
Range of A(8) vibration exposure (m/s^2^)	0—12	2–8.9

### Neurosensory and vascular symptoms

#### Comparison between quarry and foundry workers

When comparing the quarry workers with the foundry workers there was no difference for 2-PD and the needle tests. The foundry workers performed significantly better than the quarry workers for the monofilament tests in dig 2 and 5 bilaterally (*p* ≤ 0.016), TPT warmth in dig 2 (*p* = 0.048) and 5 dexter (*p* = 0.008), and in dig 5 sinister (*p* = 0.005). They also showed a better VPT performance in dig 5 dexter (*p* = 0.031).

The muscle strength tests in the right and left hand were of the same magnitude in the two groups with the exception of the pinch grip in the left hand, where the foundry workers performed better than the quarry workers (*p* = 0.014).

### Grading of vibration white fingers and neurosensory findings

None of the quarry workers were classified as vibration white fingers (Table 2). One foundry worker was classified as SWS (Stockholm Workshop Scale) stage 1. Four quarry workers showed signs of neurosensory injuries (2 were staged as SWS 2 SN and 2 as SWS 1 SN). Two foundry workers were staged as SWS 1 SN.

**Table 2:** Grading of vibration white fingers and neurosensory findings

SWS RH	Quarry w	Foundry w	SWS LH	Quarry w	Foundry w
SWS 0	10	14	SWS 0	10	14
SWS 1	0	1	SWS 1	0	1
SWS 2	0	0	SWS 2	0	0
SWS 3	0	0	SWS 3	0	0
SWS 0 SN	6	14	SWS 0 SN	7	13
SWS 1 SN	2	1	SWS 1 SN	1	2
SWS 2 SN	2	0	SWS 2 SN	2	0
SWS 3 SN	0	0	SWS 3 SN	0	0

The number of workers and the grading of VWF and neurosensory findings (SN) in their right (RH) and left hands (LH) according to the Stockholm Workshop Scale (SWS). *W *workers

### Comparison between right and left hand

The comparison between right and left hand in the total material showed no significant differences for 2-PD, tuning fork, needle test, vibration perception thresholds (VPT), Jamar, Pinch grip and 3-Chuck grip. A slight difference was noted for TPT warmth, which was significantly higher in dig 2 dexter compared to dig 2 sinister, indicating reduced sensitivity, (*p* = 0.027). The monofilament test showed a reduced sensitivity in dig 2 right hand as compared with dig 2, left hand (*p* = 0.046), while there was no difference for dig 5. A strong correlation was noted between the Pinch grip strength in the left and right hand (*r*_s_ = 0.89; *p* < 0.001).

### Musculoskeletal symptoms and diagnosis

The musculoskeletal examination of the group of quarry workers (*N* = 9) at baseline showed that four of the workers (44%) had experienced symptoms from both neck and hands during the last seven days. Some of them also had symptoms from shoulders and/or elbow. Two workers were diagnosed with tension neck syndrome and one with ulnar entrapment in elbow. Only two of the nine examined workers had been symptom-free in the neck, shoulders, elbows and hands for the past year.

## Discussion

In this study, both quarry and foundry workers had a long and high vibration exposure exceeding the exposure limit value (5 m/s^2^; Table 1). In general, the foundry workers performed better than the quarry workers, e.g. for pinch grip, monofilament tests and the TPT determinations. It is probable that the exposure to transient and high-frequency vibrations was considerably higher at the quarry, but there is not enough data from vibration measurements to evaluate this further.

A high prevalence of VWF and neurosensory findings has been reported in a number of studies on quarry and foundry workers (Hamilton 1918; Agate 1949; Starck et al. 1983; Taylor et al. 1984; Bovenzi et al. 1988,1994; Kákosy et al. 2003). Unexpectedly, despite this long-term and high vibration exposure, the prevalence of vibration white fingers in our study was considerably lower (≤ 7%) at both work-sites than in the cited studies. A higher prevalence was noted for neurosensory findings, approximately, 40% among the quarry workers and 13% among the foundry workers (Table 2). A recent systematic review and meta-analysis by Nilsson et al. (2017) shows that neurosensory injuries appear several years earlier than VWF, which is consistent with the findings in our study.

In an on-going Swedish study from a load assembly plant (Gerhardsson et al. 2020), 38 vibration exposed workers (30 males, 8 females) were studied. The prevalence of VWF was 30% among the male workers and 50% among the females. The corresponding prevalence of neurosensory symptoms was 70% among the males and 88% among the females, despite self-estimated A(8) values of 2.2 and 1.8 m/s^2^ in the right and left hand, respectively. These values are clearly below the current action limit value of 2.5 m/s^2^. Transient and high-frequency vibrations from the tools used are stretching the arteries, nerves and corpuscles in a repetitive way and the shock wave that propagates into the tissues may not give the tissues enough time to recover. Shock wave vibration exposure may thus lead to a progressive cell death of sensory neurons and Schwann cells and hamper the regeneration of damaged nerve endings which has been shown in animal experiments on rats (Zimmerman et al. 2020).

In an Italian study of 76 stonedrillers and stonecutters/chippers, 27 subjects (36%) had developed VWF (Bovenzi et al. 1988) with a median latency time of 10 years. High vibration levels (ISO 5349-1) between 20 and 36 m/s^2^ were noted for rock drills and chipping hammers and there was a significant relationship between the vibration exposure level and the stage of VWF. In another study of vibration-exposed workers Bovenzi et al. (1998) studied 570 quarry drillers and 258 control stone workers, who performed only manual activity. The prevalence of vibration white fingers was 30% and the prevalence of neurosensory findings was 40% among the vibration-exposed workers. During stone drilling and breaking, the average frequency weighted acceleration in the dominant axis was 15 m/s^2^. In a subgroup, 41 quarry drillers had a mean A(8) value of 8.3 m/s^2^ and a total operating time of 18 600 h. Another subgroup included 31 foundry workers who had an A(8) value of 4.7 m/s^2^ and a total exposure time of 15 400 h. These A(8) values are comparable to the values in our study but the exposure times are longer. The prevalence of VWF in these two subgroups was 37% among the quarry drillers and 52% among the foundry workers (Bovenzi et al. 1998).

Agate (1949) studied 278 workers (45 women), who were polishing metal castings with rotary hand tools. In this group, 70% of the 233 males and 47% of the 45 females developed vibration white fingers. The average time between the start of the polishing work and the appearance of the first symptoms was no longer than 24 months in males and 21 months in females.

When Taylor et al. (1984) revisited the quarries in the Bedford Indiana area in 1978, 60 years had passed since the studies of Hamilton (1918). The latter examined 123 workers in three branches of soft stone, marble, and granite work and found only 17 subjects who did not show signs of so-called dead fingers. Taylor et al. (1984) examined 30 remaining long-term exposed subjects and found a high prevalence of VWF (63%). The highest prevalence (80%) was observed among 15 stonecutter/carvers using a light 3–4 lb hammer. In the total material, neurosensory findings affecting touch and TPT were found in 75% of the subjects in stage 3 and in about 55% in stage 2. Similar findings have been reported by Telford et al. (1945). He examined engineering workers, mainly polishers and cutters, in northern England, who showed a prevalence of VWF of about 25%. The left hand had the highest exposure and was often the only hand to be affected. In our study, however, only minor differences were noted between left and right hand as regards both VWF and neurosensory symptoms and signs.

The tool grip will have an influence on the vibration transmission from tool to hand. In a Finnish study (Färkkilä et al. 1978), 16 vibration-exposed workers using pneumatic hammers were studied. The right hand held the barrel of the hammer and the left hand held the chisel head, which gave the highest vibration exposure. Of the 16 workers investigated, 14 developed vibration white fingers. The symptoms started in the left hand in nine subjects. Eleven workers participated in a cold provocation test and for seven of them the result was positive. For six of them the left hand was most severely affected.

Simultaneous exposure to cold can increase the risk of vibration white fingers as it causes a further decrease of the blood flow in the finger vessels, which aggravates the condition. In parallel, the skin temperature in the hands/fingers is decreased even further, which increases the VWF symptoms. Reductions in blood flow can probably also worsen the neurosensory symptoms.

The musculoskeletal examination of the quarry workers in our study showed a high prevalence of symptoms in both neck and hands. The prevalence of symptoms was comparable to the highest prevalence rates reported from examinations of other groups with the HECO-method, e.g. butchers and molders. The limited number of quarry workers, however, makes the comparison uncertain at a group level.

The high prevalence of neck symptoms is probably explained by a working posture with the head bent forward during work to see the position of the drill in combination with lifting and handling of the heavy vibrating machines. Prolonged exposure to neck flexion is considered as a risk factor for neck pain (Ariens et al. 2001; Palmer and Smedley 2007; Mayer et al. 2012).

The vibration exposure and the power grips needed to stabilize the vibrating tools could contribute to the hand symptoms. Biomechanical load, especially in combination with repetitive and forceful work is a risk factor for hand and wrist disorders (Malchaire et al. 1997).

Also, other factors must be considered when discussing vibration injuries, e.g. the characteristics of the vibration (magnitude, frequency, duration), the working method, environmental conditions, stress factors and individual susceptibility. Furthermore, the individual exposure estimation is also dependent on the psychosocial disposition of the worker, which in turn may contribute to an overestimate or underestimate of exposure time. Other factors of importance beside diagnostic uncertainties are the biological variation and the healthy worker effect. Sensitive subjects may leave work early, while workers staying for many years may be a population of survivors. Self-assessment of exposure time can introduce a considerable variation between workers doing the same work task.

A high prevalence of VWF and neurosensory findings has been reported in a number of studies on quarry and foundry workers. The prevalence varies a lot among different studies but the peak values reach 70–100% for VWF and 65–100% for neurosensory findings (Kákosy et al. 2003; Starck et al. 1983; Agate 1949; Hamilton 1918; Taylor et al. 1984; Bovenzi et al. 1988,1994). The low prevalence of VWF in our study is difficult to explain as the vibration exposure was clearly exceeding the current exposure limit value of 5 m/s^2^ with quite long exposure times. Furthermore, despite the fact that the quarry workers worked outdoors and were exposed to cold as well as to hand-arm vibrations during the winter period, their prevalence of VWF was very low compared to their prevalence of neurosensory findings (40%). The same tendency has been published recently, in a study from a loader assembly plant in Sweden (Gerhardsson et al. 2020) where the workers had a high exposure to high-frequency vibrations. Thus, several studies indicate that high-frequency vibrations increase the risk for developing neurosensory symptoms and that the nervous system may be more susceptible to exposure to high-frequency vibrations than the vascular system. It is important to consider that the ISO 5349-1 standard is not applicable for the evaluation of neurosensory injuries caused by hand-arm vibration. Neurosensory injuries appear earlier than vascular injuries (Nilsson et al. 2017), are more disabling and have a worse prognosis.

The ISO 5349-1 standard is the basis for the estimation of hand-arm vibration exposure. Vibrations measured according to the standard are frequency weighted and band limited to 1250 Hz. For evaluation of health effects the standard includes a relationship between the daily vibration exposure, A(8)-value, the group mean exposure duration in years and a 10% prevalence of vibration white finger (VWF).

The ISO 5349-1 standard contains no information about a relationship between the prevalence of neurosensory injury and vibration exposure. One of the results in the systematic review by Nilsson et al. (2017) is that neurosensory injury latency is shorter than the corresponding latency for VWF, for the same vibration exposure. Thus, health effects from hand-arm vibration exposure developed faster than indicated in the standard. The high prevalence of neurosensory injuries and the low prevalence of VWF in our study seem to comply better with the health – exposure relationship presented in the systematic review by Nilsson et al. (2017) than with the corresponding relationship in the ISO 5349–1 standard.

A high prevalence of HAVS among workers using impacting or high-speed tools has been observed despite low or very low daily vibration exposure when evaluated according to the current standard (Gerhardsson et al. 2020). The reason for that is not fully clarified. Impacting and high-speed tools generate high vibration amplitudes in the frequency range above the ISO standard band limiting filter. The impact of the high-frequency content may be part of the explanation to the high prevalence of HAVS despite low A(8) values. For impacting tools, the damage could be caused by the impact force and accordingly a time-domain approach could be more appropriate for the evaluation.

It is not clear to what extent impacting or high-speed tools contribute to the shorter latency for neurosensory injuries found in the systematic review by Nilsson et al. (2017). If damage to the Aβ, Aδ and C nerve fibres are more likely to occur due to exposure to high-frequency vibrations or impacts, it will affect the A(8) – HAVS prevalence relationship. That is, the current A(8) value may not be representative for the health risk estimation, especially for neurosensory injuries.

To enable a full health risk assessment for vibration exposure, the ISO 5349-

1 standard must be updated and include a relationship between neurosensory injuries and vibration exposure. The importance of high-frequency vibrations and impacts should be investigated as studies (Gerhardsson et al. 2020) indicate a poor correlation between the current A(8) value and neurosensory injuries.

## Conclusions

Despite the high exposure exceeding the exposure limit value of 5 m/s^2^ the prevalence of VWF was low in both groups. The high prevalence of neurosensory findings among the quarry workers may depend on a higher A(8) vibration exposure, longer exposure time, longer work periods with continuous vibration exposure and a higher exposure to high-frequency vibrations compared to the foundry workers. Furthermore, there is probably also a contributing age factor as the quarry workers were 8.5 years older (mean value) than the foundry workers. Another contributing factor is exposure to cold as the quarry workers mainly work outdoors, in a cold environment during the winter period, while the foundry workers work indoors.

## Data Availability

The datasets used and/or analysed during the current study are available from the corresponding author on reasonable request.
